# Comparison of ultrasound-guided two-point block of the rhomboid intercostal *vs.* thoracic paravertebral for postoperative analgesia in patients undergoing three-port thoracoscopic surgery: a prospective, randomized, non-inferiority study

**DOI:** 10.1080/07853890.2026.2713836

**Published:** 2026-08-08

**Authors:** Mingzi An, Yueru Hou, Chengyun Xu, Zhenping Li, Yazhi Xi, Xiaoyu Jia, Lei Xie, Qinghe Zhou

**Affiliations:** ^a^ Department of Anesthesiology and Pain Medicine, Affiliated Hospital of Jiaxing University, Jiaxing, China; ^b^ Department of Anesthesiology and Pain Medicine, The Second Affiliated Hospital of Jiaxing University, Jiaxing, China

**Keywords:** Rhomboid intercostal block, thoracoscopic surgery, thoracic paravertebral block, ultrasound-guided, postoperative analgesia

## Abstract

**Background:**

The anesthetic characteristics of ultrasound-guided two-point rhomboid intercostal block (RIB) have not been fully established. This study compared the analgesic efficacy of ultrasound-guided two-point RIB with that of two-point thoracic paravertebral block (TPVB) for postoperative pain control and recovery in patients undergoing three-port thoracoscopic surgery.

**Methods:**

Seventy patients aged 18–80 years scheduled for three-port thoracoscopic pulmonary resection were block-randomized in a 1:1 ratio to receive either TPVB or RIB. Both techniques were performed using 10 mL of 0.5% ropivacaine at each injection site. The primary outcome was the numerical rating scale (NRS) pain score at rest 24 h after surgery, with a predefined non-inferiority margin of Δ = 1. Secondary outcomes included NRS at rest and during coughing at 0.5, 2, 4, 12, 18, 24, and 48 h postoperatively, as well as the 24h postoperative Quality of Recovery-40 (QoR-40) score.

**Results:**

The final analysis included 34 patients in the TPVB group and 34 in the RIB group. The mean difference in resting NRS scores at 24 h between the two groups was 0.088 (95% CI, −0.377 to 0.553), confirming the non-inferiority of RIB. However, the need for rescue analgesia was numerically greater in the RIB group than in the TPVB group (*p* = 0.045). The 24-h postoperative QoR-40 scores and cumulative sufentanil consumption within 48 h after surgery were comparable between the groups (both *p* > 0.05).

**Conclusion:**

Ultrasound-guided two-point RIB provided postoperative analgesia that was non-inferior to TPVB in patients undergoing three-port thoracoscopic surgery.

## Highlights

- Postoperative pain after minimally invasive thoracoscopic surgery is unavoidable. While ultrasound-guided paravertebral nerve block is the gold standard for analgesia, safety concerns remain.
- The effectiveness of a single ultrasound-guided rhomboid intercostal block for pain relief following thoracoscopic surgery is well-documented, although its efficacy in three-port procedures remains unclear.
- This randomized controlled study confirmed that the analgesic efficacy of ultrasound-guided two-point rhomboid intercostal block in three-port thoracoscopic surgery is not inferior to that of paravertebral nerve block.

## Graphical abstract

**Figure:**
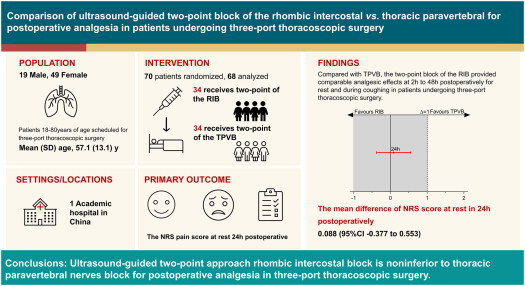

## 1. Introduction

Video-assisted thoracoscopic surgery (VATS) has become a widely adopted alternative to open thoracotomy because it is associated with less surgical trauma and faster recovery [1,2]. However, despite its minimally invasive nature, multiport VATS is still associated with considerable postoperative pain, particularly during early mobilization [3,4]. Effective perioperative pain management is therefore essential to facilitate recovery, reduce opioid consumption and its associated adverse effects, and improve postoperative outcomes. Regional nerve blocks provide rapid, long-lasting analgesia and have become an integral component of multimodal analgesic strategies for thoracic surgery.

Thoracic epidural anesthesia (TEA) has long been regarded as the gold standard for analgesia after thoracic surgery. However, its relatively high incidence of adverse effects, including hypotension and urinary retention, limits its compatibility with enhanced recovery after surgery (ERAS) protocols. Thoracic paravertebral block (TPVB) offers effective postoperative analgesia after thoracoscopic surgery and has emerged as a suitable alternative to TEA for managing post-thoracoscopic pain [5]. Despite its clinical effectiveness, TPVB presents technical challenges because the needle is advanced close to the pleura, increasing the risk of complications such as hypotension, chest wall hematoma, pneumothorax, and inadvertent epidural spread [6].

The rhomboid intercostal block (RIB) is a relatively novel fascial plane block for chest wall analgesia that provides sensory coverage of the surgical incision while positioning the injection site away from the thoracic cavity and major vascular structures [7–9]. Our previous study demonstrated that a single-shot RIB provided postoperative recovery outcomes that were non-inferior to those achieved with a single-shot TPVB following thoracoscopic surgery [10]. However, three-port thoracoscopic surgery involves multiple incisions across different thoracic dermatomes, and it remains uncertain whether a single injection can provide adequate analgesic coverage. Previous studies have shown that local anesthetic injected into the fascial plane between the rhomboid major and intercostal muscles can spread from T2 to T9 [11], and a two-point injection technique may further improve anesthetic distribution across the three-port surgical field.

It was, therefore, hypothesized that ultrasound-guided two-point RIB would provide postoperative analgesia and facilitate postoperative recovery comparable to those achieved with ultrasound-guided two-point TPVB in patients undergoing three-port thoracoscopic surgery.

## 2. Methods

### 2.1. Study participants

This study was approved by the Ethics Committee of the Affiliated Hospital of Jiaxing University (2023-KY-340) and registered with the Chinese Clinical Trial Registry (ChiCTR2300073661). Written informed consent was obtained from all participants before enrollment. The study was conducted in accordance with the principles of the Declaration of Helsinki and Good Clinical Practice guidelines. Patients scheduled to undergo elective thoracoscopic lung segmentectomy at the Affiliated Hospital of Jiaxing University between January 4, 2024, and December 31, 2024, were prospectively enrolled. The study protocol and statistical analysis plan are provided in the Supplementary Materials 1.

The inclusion criteria were as follows: (1) patients scheduled to undergo unilateral three-port thoracoscopic surgery; (2) age between 18 and 80 years; and (3) American Society of Anesthesiologists (ASA) physical status I–III. The exclusion criteria were: (1) significant cardiac, pulmonary, hepatic, renal, or coagulation dysfunction (left ventricular ejection fraction ≤40%, PaO_2_/FiO_2_ ratio ≤300 mmHg, Child–Pugh class C, serum creatinine ≥133 μmol/L, or international normalized ratio [INR] ≥ 1.2); (2) infection at the planned block site or severe nerve injury involving the ipsilateral upper limb; (3) a history of chronic analgesic or psychiatric medication use, or contraindications to nonsteroidal anti-inflammatory drugs; (4) allergy to local anesthetics, including lidocaine or ropivacaine, or to general anesthetic agents; (5) refusal of surgery or anesthesia by the patient or their legal representative; and (6) inability to complete the study questionnaires.

The predefined withdrawal criteria were: (1) occurrence of serious intraoperative complications; (2) intraoperative blood loss exceeding 800 mL; (3) withdrawal of consent or refusal to participate in postoperative follow-up; and (4) conversion from three-port thoracoscopic surgery to either open thoracotomy or single-port thoracoscopic surgery.

## 3. Study design

### 3.2. General anesthesia and postoperative management

General anesthesia was administered using a balanced intravenous and inhalational technique. Anesthesia was induced with rocuronium (0.6 mg/kg), sufentanil (0.4–0.8 μg/kg), propofol (2 mg/kg), and atropine (0.5 mg). Anesthesia was maintained with a continuous infusion of propofol at 50–150 μg/(kg·min), remifentanil at 0.1–1 μg/(kg·min), and inhaled sevoflurane at 1.0–1.5 minimum alveolar concentration (MAC). Additional boluses of rocuronium (0.15 mg/kg) were administered as needed throughout the procedure to maintain adequate neuromuscular blockade according to the drug’s pharmacokinetics and intraoperative clinical requirements. A 35-Fr or 37-Fr double-lumen endotracheal tube was selected according to the patient’s sex. All patients underwent volume-controlled mechanical ventilation with a tidal volume of 6–8 mL/kg ideal body weight, a respiratory rate of 10–16 breaths/min, and end-tidal carbon dioxide (PetCO_2_) maintained between 35 and 45 mmHg.

Anesthetic depth was continuously monitored using an Entropy Sensor (GE Healthcare Finland Oy, Finland) and adjusted accordingly. Intraoperative fluid therapy followed the 4–2–1 rule to maintain hemodynamic stability, and vasoactive agents were administered when clinically indicated to manage hemodynamic fluctuations.

During skin closure, ondansetron (4 mg) was administered intravenously for prophylaxis against postoperative nausea and vomiting (PONV). At the end of surgery, patients were connected to a patient-controlled intravenous analgesia (PCIA) pump containing sufentanil 100 μg diluted with 0.9% sodium chloride to a total volume of 100 mL. The PCIA was programmed to deliver a 2-mL bolus on demand, with a 15-minute lockout interval, an initial loading dose of 2 mL, and no continuous background infusion. During the first 48 h after surgery, patients with an NRS pain score ≥4 despite activating the PCIA received intravenous parecoxib 40 mg as rescue analgesia, with a maximum daily dose of 80 mg.

### 3.3. Regional blocks

#### 3.3.1. TPVB group

Patients assigned to the TPVB group received ultrasound-guided two-point thoracic paravertebral blocks at the T5 and T7 levels. Using a high-frequency linear ultrasound transducer (10 MHz; LOGIQ e, GE Healthcare, Deutschland GmbH & Co. KG, Solingen, Germany), the probe was initially positioned in a parasagittal orientation at the T5 level adjacent to the midline to identify the spinous process. The probe was then moved laterally toward the surgical side until the transverse process, pleura, and internal intercostal membrane were clearly visualized. Under real-time ultrasound guidance, the block needle was advanced in-plane toward the paravertebral space until its tip reached the base of the transverse process. After correct needle placement was confirmed by pleural displacement, 10 mL of 0.5% ropivacaine was injected, with ultrasound visualization of local anesthetic spread within the paravertebral space. The same procedure was subsequently performed at the T7 level (S2-eFigure 1 in the Supplementary Materials 2).

#### 3.3.2. RIB group

**Figure 1.**
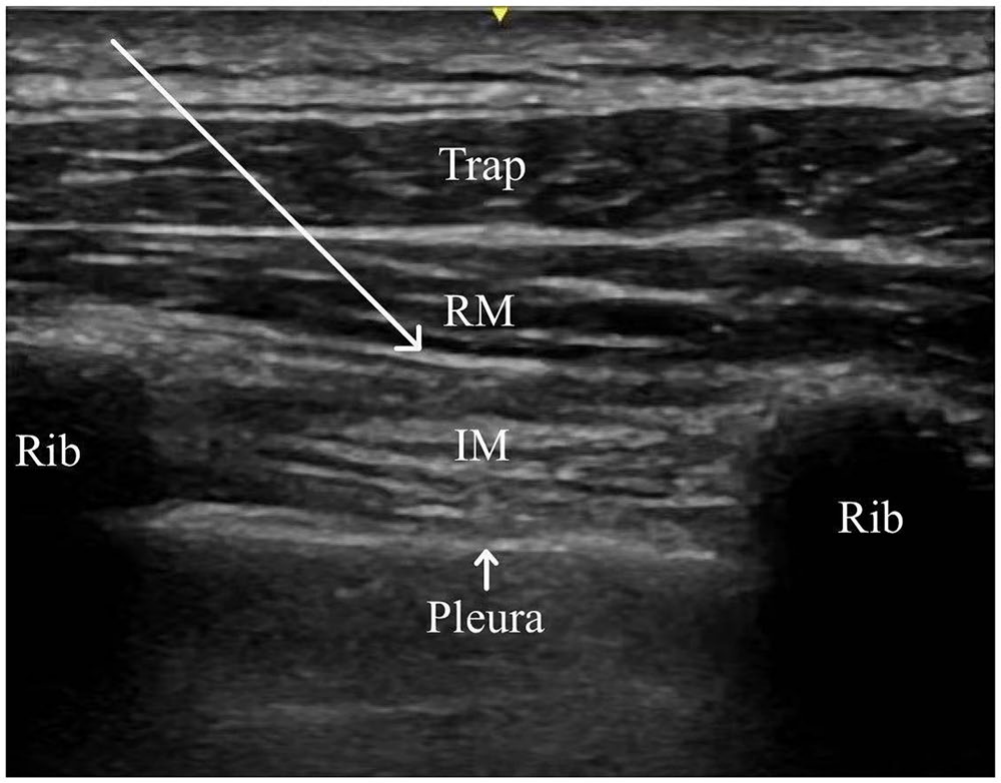
Diagram illustrating ultrasound guidance of rhomboid intercostal block. Trap: trapezius. RM: Rhomboid muscle, IM: Intercostal muscle. The white arrow indicates the local anesthetic injection site for needle puncture.

Patients assigned to the RIB group were placed in the lateral decubitus position with the ipsilateral arm adducted across the chest to facilitate lateral displacement of the scapula. A high-frequency linear ultrasound transducer (10 MHz) was positioned in an oblique parasagittal plane between the T4 and T5 vertebral levels and moved medially along the medial border of the scapula on the surgical side. The trapezius, rhomboid major, intercostal muscles, pleura, and lung were identified as anatomical landmarks. Under ultrasound guidance, the block needle was advanced in-plane into the fascial plane between the rhomboid major and intercostal muscles. After injection of 0.5–1 mL of 0.5% ropivacaine to confirm correct hydrodissection of the fascial plane, an additional 10 mL of 0.5% ropivacaine was administered. An identical procedure was then performed between the T6 and T7 levels using the same technique (Figure 1).

### 3.4. Randomization and blinding

Before patient enrollment, an investigator who was not involved in the conduct of the study generated the randomization sequence using block randomization with a 1:1 allocation ratio and a block size of four. Group assignments were sealed in sequentially numbered, opaque envelopes. On the day of surgery, a separate investigator opened the next envelope in sequence and assigned each patient to the corresponding study group. Patients, surgeons, outcome assessors, and statisticians were all blinded to treatment allocation. Ultrasound-guided regional blocks were performed in the pre-anesthesia preparation area by an experienced anesthesiologist who was not involved in postoperative follow-up or data analysis.

### 3.5. Outcomes

Patient demographic characteristics, surgical details, and anesthetic data were collected from the electronic anesthesia record system and patients’ medical records. The primary outcome was the NRS pain score at rest 24 h after surgery. Secondary outcomes included NRS pain scores at rest and during coughing at 0.5, 2, 4, 12, 18, 24, and 48 h postoperatively, the Quality of Recovery-40 (QoR-40) score at 24 h, cumulative sufentanil consumption during the first 48 h after surgery, and the proportion of patients requiring rescue analgesia within 48 h postoperatively. Safety outcomes assessed within the first 24 h after surgery included PONV, hypotension, chest wall hematoma, respiratory depression, shivering, urinary retention, and inadvertent epidural anesthesia. Postoperative pulmonary complications (PPCs) occurring within 7 days after surgery included pneumothorax, pleural effusion (>300 mL), atelectasis, hypoxemia, pulmonary infection, and pneumonia. Detailed definitions of these pulmonary complications are provided in the Supplementary Materials 1.

### 3.6. Statistical analysis

A pilot study involving 20 patients (unpublished data) demonstrated that the mean resting NRS pain score at 24 h after surgery was 2.40 ± 0.70 in the RIB group and 2.30 ± 1.25 in the TPVB group (control). Statisticians and clinical experts generally recommend defining the non-inferiority margin (Δ) as 20%–50% of the standard deviation of the control group. The minimal clinically important difference (MCID) for acute postoperative pain measured using the visual analog scale (VAS) or NRS is 1 point [12]. Previous non-inferiority trials evaluating postoperative analgesia following thoracoscopic surgery have also adopted a non-inferiority margin of 1 [13]. Because NRS scores are recorded as whole integers, a non-inferiority margin of Δ = 1 was considered clinically appropriate.

Based on a two-sample non-inferiority test for independent means, a minimum of 56 evaluable patients was required to achieve 90% power with a one-sided α of 0.025. To allow for an anticipated dropout rate of 20%, 70 patients were scheduled for enrollment. Non-inferiority was established if the upper limit of the 95% confidence interval (CI) for the between-group difference in the mean resting NRS score at 24 h did not exceed the predefined margin of Δ = 1. The between-group differences in NRS scores at all other postoperative time points within the first 48 h were evaluated using the same non-inferiority criterion (Δ = 1).

Baseline characteristics were summarized using descriptive statistics. Normally distributed continuous variables are presented as the mean ± standard deviation (SD), whereas non-normally distributed variables are expressed as the median (interquartile range [IQR]). Categorical variables are reported as frequencies and percentages. Between-group comparisons were performed using the independent-samples t test or the Mann–Whitney U test for continuous variables, and the chi-square test or Fisher’s exact test for categorical variables, as appropriate. Missing data were handled using multiple imputation. All statistical analyses were performed with SPSS version 25.0 (IBM Corp., Armonk, NY, USA), and figures were generated using GraphPad Prism version 9. A two-sided *p* < 0.05 was considered statistically significant.

## 4. Results

### 4.1. Patient characteristics

**Figure 2.**
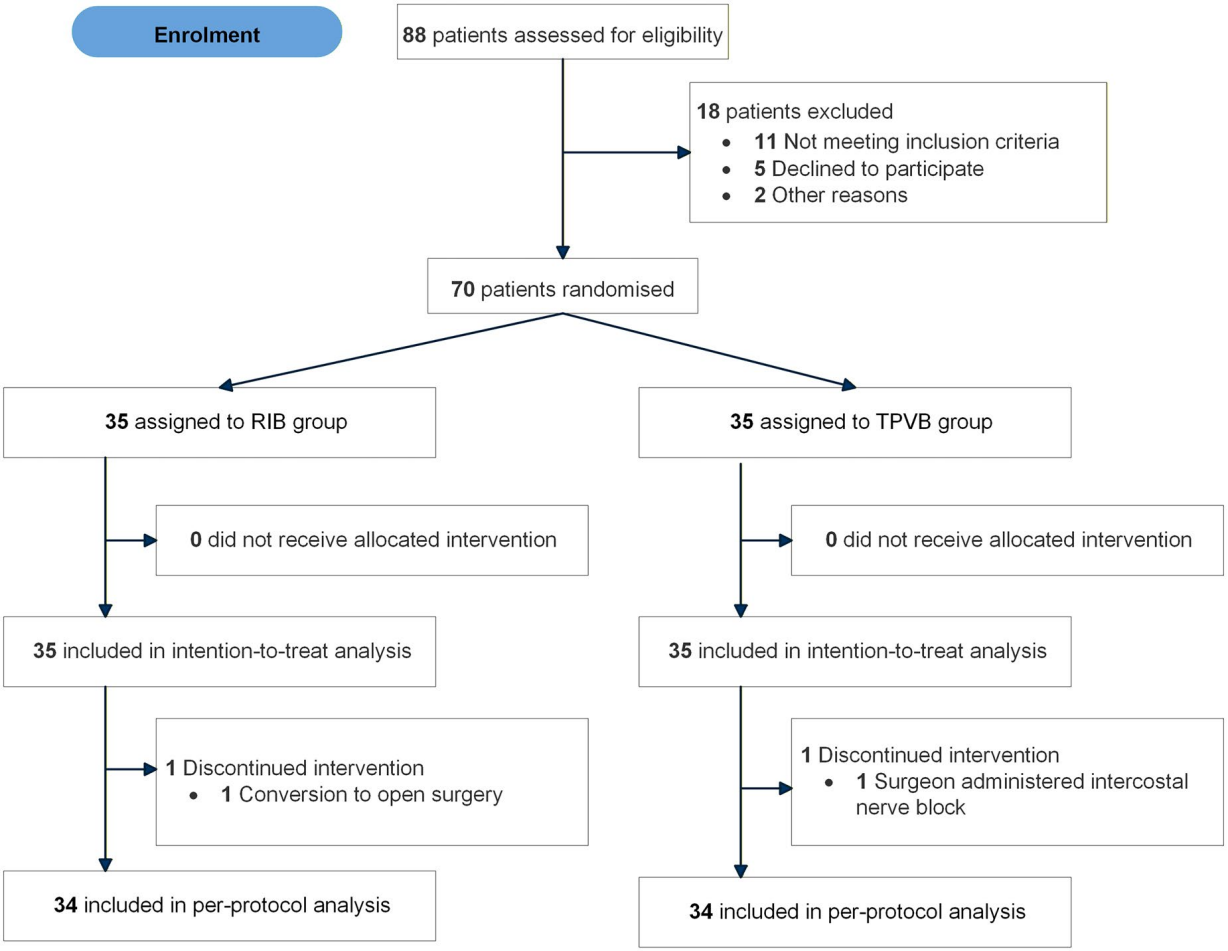
CONSORT diagram.

Between January 4, 2024, and December 31, 2024, a total of 88 patients were screened for eligibility. Of these, 70 met the inclusion criteria, were enrolled, and were randomized to the study groups. All participants received the assigned analgesic intervention according to the study protocol (Figure 2). One patient in the TPVB group was excluded from the per-protocol (PP) analysis because an additional intercostal nerve block was administered by the surgeon after surgery, and one patient in the RIB group was excluded because the thoracoscopic procedure was converted to open thoracotomy intraoperatively.

All enrolled patients completed the 48-h postoperative follow-up, including NRS pain assessments through 48 h, the 24-h QoR-40 evaluation, and postoperative safety assessments. Therefore, no missing data were identified, and no data imputation was required. The intention-to-treat (ITT) analysis included all 70 randomized patients (35 per group), whereas the PP analysis included 68 patients (34 per group).

Baseline demographic and clinical characteristics were comparable between the two groups, with no statistically significant differences observed in either the ITT or PP populations (all *p* > 0.05) (Table 1 and S2-eTable 1). Operative side, anesthetic management, operative duration, and intraoperative anesthetic drug consumption did not differ significantly between the RIB and TPVB groups in either analysis population (all *p* > 0.05) (S2-eTable 1 and S2-eTable 2 in the Supplementary Materials 2).

**Table 1.** Patient characteristics and clinical data.

	RIB (*n* = 34)	TPVB (*n* = 34)
Age, y	57.3 ± 13.9	56.9 ± 12.4
Height, cm	160.0(155.0–168.0)	160.0(157.0–163.0)
Weight, kg	61.60(53.9–67.5)	61.40(54.0–66.8)
Sex, Male/Female	12/22	7/27
Lung surgery history, n%	4(11.8%)	3(8.8%)
ASA, n%		
I	1(2.9%)	0(0.0%)
II	30(88.2%)	33(94.1%)
III	3(8.8%)	1(2.9%)
Pathology. n%		
Malignant	31(91.2%)	33(97.1%)
Benign	3(8.8%)	1(2.9%)
Comorbidities. n%		
Hypertension	12 (35.29%)	12 (35.29%)
Diabetes	4 (11.76%)	3 (8.82%)
Stroke	1 (2.94%)	0 (0.00%)
Immunometabolic	3 (8.82%)	1 (2.94%)
COPD	1 (2.94%)	0 (0.00%)
Liver disease	1 (2.94%)	2 (5.88%)
Depression	1 (2.94%)	2 (5.88%)

Notes: ASA, American Society of Anesthesiologists; COPD, chronic obstructive pulmonary disease.

The results presented in the main manuscript are based on the PP analysis, whereas the findings from the ITT analysis are provided in the Supplementary Materials 2.

### 4.2. Primary outcome

**Figure 3.**
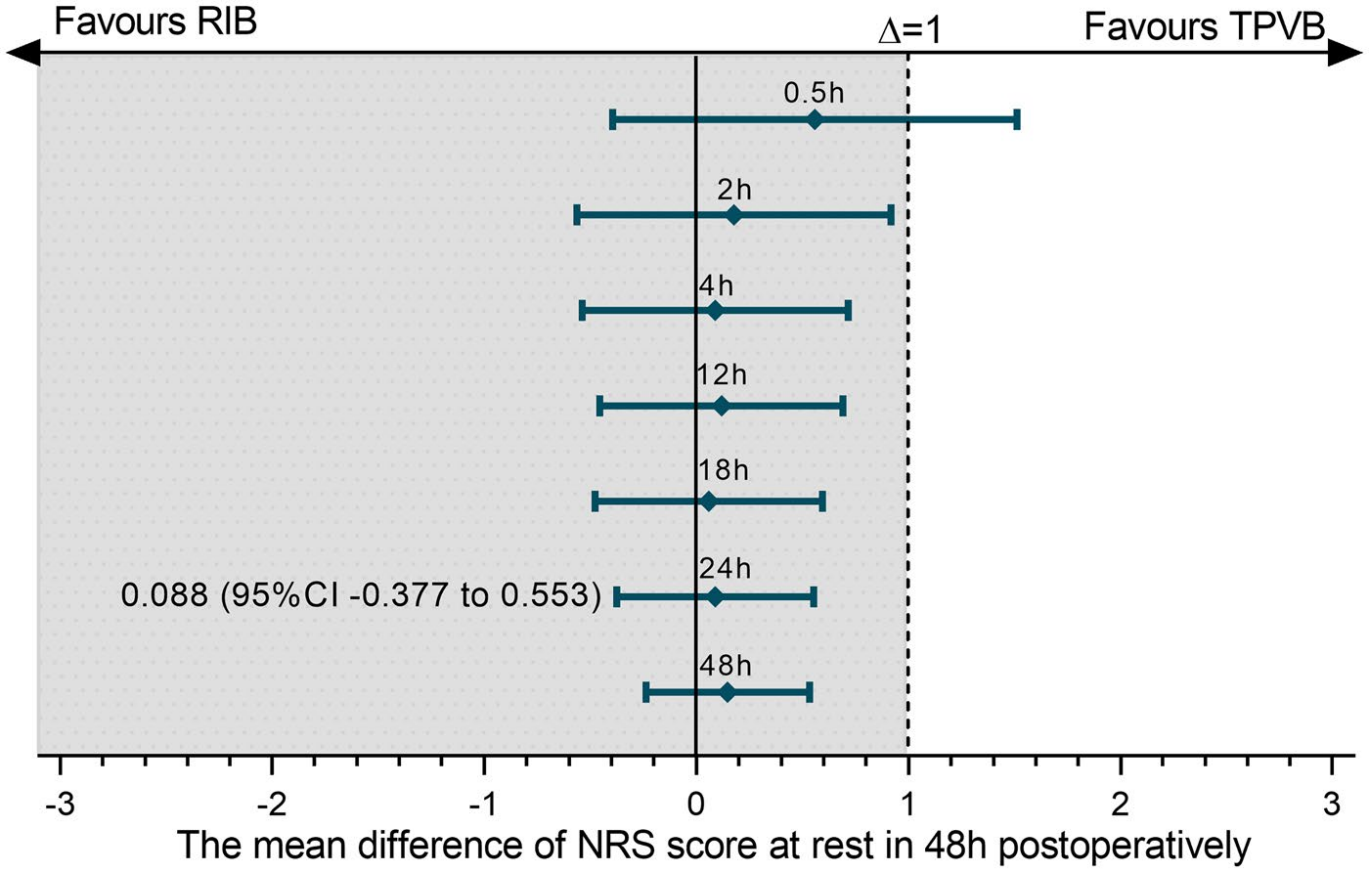
Non-inferiority comparison of NRS scores measured at rest within 48 h of RIB and TPVB procedures. RIB: Rhomboid intercostal block, TPVB: Thoracic paravertebral block, Δ: Non-inferiority margin, NRS: Numerical rating scale, CI: Confidence interval.

The mean between-group difference in resting NRS pain scores at 24 h after surgery (RIB minus TPVB) was 0.088 (95% CI, −0.377 to 0.553). Because the upper limit of the 95% confidence interval (0.553) was below the predefined non-inferiority margin of 1, RIB was demonstrated to be non-inferior to TPVB for postoperative analgesia at the primary endpoint (Figure 3). The results of the ITT analysis for resting NRS pain scores during the first 48 h after surgery are presented in S2-eFigure 2 in the Supplementary Materials 2.

### 4.3. Secondary outcome

Resting NRS pain scores at 2, 4, 12, 18, and 48 h after surgery demonstrated the non-inferiority of RIB compared with TPVB. The mean between-group differences (RIB minus TPVB) at these time points were 0.177 (95% CI, −0.565 to 0.918), 0.088 (95% CI, −0.539 to 0.716), 0.118 (95% CI, −0.457 to 0.692), 0.059 (95% CI, −0.477 to 0.595), and 0.147 (95% CI, −0.238 to 0.533), respectively. In all cases, the upper limit of the 95% confidence interval remained below the predefined non-inferiority margin (Δ = 1), confirming non-inferiority (Figure 3). In comparison, at 0.5 h after surgery, the upper limit of the 95% confidence interval exceeded the non-inferiority margin, with a mean difference of 0.556 (95% CI, −0.395 to 1.512). Corresponding results from the ITT analysis are presented in S2-eFigure 2 in the Supplementary Materials 2.

NRS pain scores during coughing at 2, 4, 12, 18, 24, and 48 h after surgery confirmed the non-inferiority of RIB to TPVB. The mean between-group differences were 0.147 (95% CI, −0.559 to 0.892), 0.059 (95% CI, −0.589 to 0.707), 0.177 (95% CI, −0.497 to 0.850), 0.000 (95% CI, −0.583 to 0.583), 0.206 (95% CI, −0.263 to 0.675), and 0.206 (95% CI, −0.195 to 0.607), respectively. None of the upper confidence limits exceeded the predefined non-inferiority margin (Δ = 1), as shown in S2-eFigure 3 and S2-eFigure 4. However, at 0.5 h postoperatively, the upper limit of the 95% confidence interval was greater than 1, with a mean difference of 0.530 (95% CI, −0.451 to 1.510).

Total sufentanil consumption through the patient-controlled intravenous analgesia pump during the first 48 h after surgery did not differ significantly between the two groups (*p* > 0.05). However, patients in the RIB group required rescue analgesia more frequently than those in the TPVB group (*p* = 0.045) (Table 2). The 24-h postoperative QoR-40 scores were comparable between the groups (RIB vs. TPVB: 180.4 ± 6.1 vs. 180.4 ± 6.3, *p* = 0.969) (Table 3). The ITT analysis yielded findings consistent with those of the PP analysis and is presented in S2-eTable 3 and S2-eTable 4 in the Supplementary Materials 2.

**Table 2.** Postoperative analgesia, adverse reactions, and complications.

	RIB (*n* = 34)	TPVB (*n* = 34)	*P-*value
Sufentanil, median (IQR), μg	16.0 (8.0–19.5)	8.0 (8.0–17.8)	0.455
Rescue analgesia, n%	12 (35.3%)	4 (11.8%)	0.045
Postoperative complications, n%	7 (20.6%)	12 (35.3%)	0.177
PONV	6 (17.65)	8 (23.53%)	0.549
Hypotension	0 (0.0%)	2 (5.88%)	0.473
Chills	1 (2.94%)	4 (11.76%)	0.353
PPCs, n%	16 (47.06%)	14 (41.18%)	0.625
Pulmonary infection	11 (32.35%)	8 (23.53%)	0.417
Pleural effusion	2 (5.88%)	5 (14.71%)	0.425
Atelectasis	7 (20.59%)	5 (14.71%)	0.525
Hypoxemia	0 (0.00%)	1 (2.94%)	0.999
Pneumothorax	1 (2.94%)	0 (0.00%)	0.999
Pneumonia	0 (0.00%)	2 (5.88%)	0.473
Pulmonary embolism	0 (0.00%)	1 (2.94%)	0.999

Note: Rescue analgesia, the number of patients requiring rescue analgesia. PONV, postoperative nausea and vomiting; PPCs, postoperative pulmonary complications. Pulmonary complications within 7 days of surgery included pneumothorax, pleural effusion (>300 mL), atelectasis, hypoxemia, pulmonary infection, pneumonia, and pulmonary embolism.

**Table 3.** QoR-40 Scores at 24 h after surgery.

	RIB (*n* = 34)	TPVB (*n* = 34)	*P*-value
QoR-40 score at 24 h after surgery	180.4 ± 6.1	180.4 ± 6.3	0.969
Emotions	40.0 (40.0–41.0)	40.0 (40.0–41.0)	0.835
Physical comfort	55.0 (52.0–55.0)	54.0 (51.3–55.0)	0.353
Physical independence	21.0 (20.0–22.0)	20.0 (20.0–23.0)	0.818
Pain	31.5 (30.0–33.0)	33.0 (30.0–33.0)	0.300
Psychological support	34.0 (33.0–34.8)	34.0 (33.0–35.0)	0.480

Note: QoR-40, Quality of Recovery-40.

### 4.4. Safety outcomes

The overall incidence of postoperative complications was comparable between the RIB and TPVB groups (20.6% vs. 35.3%, *p* = 0.177). No cases of respiratory depression or urinary retention were observed in either group. The incidence of PPCs within 7 days after surgery did not differ significantly between the RIB and TPVB groups (47.06% vs. 41.18%, *p* = 0.625) (Table 2).

## 5. Discussion

In this prospective, randomized, non-inferiority trial, ultrasound-guided two-point RIB provided postoperative analgesia that was non-inferior to two-point TPVB in patients undergoing three-port thoracoscopic surgery. From 2 to 48 h after surgery, pain scores at rest and during coughing were comparable between the two groups. Similarly, postoperative recovery, as assessed by the QoR-40 questionnaire at 24 h, did not differ significantly, indicating that both techniques achieved similar overall recovery profiles.

Previous studies have shown that a single-shot RIB is non-inferior to a single-shot TPVB for postoperative analgesia following thoracoscopic surgery. The analgesic effect of RIB is attributed to the spread of local anesthetic within the fascial plane between the rhomboid major and intercostal muscles, where it blocks the dorsal rami of the thoracic spinal nerves and the lateral cutaneous branches of the intercostal nerves, providing analgesia to both the posterior and lateral chest wall [13,14]. Subsequent investigations further confirmed that RIB can provide sustained postoperative analgesia after thoracoscopic surgery [8,9,15]. In this study, the two-point RIB technique produced analgesia comparable to that of two-point TPVB from 2 to 48 h after surgery. These findings are consistent with previous reports and suggest that the two-point injection strategy provides sufficient anesthetic spread across the T4–T8 dermatomes, offering adequate coverage for the multiple incisions used in three-port thoracoscopic procedures [16,17].

An important finding of this study was that patients receiving RIB required rescue analgesia more frequently than those receiving TPVB, particularly during the immediate postoperative period. This observation was accompanied by inferior analgesic efficacy at 0.5 h after surgery. Several hypotheses may explain these findings. First, although injections were performed at the T4–T5 and T6–T7 levels, the volume of local anesthetic administered may not have spread sufficiently within the fascial plane between the rhomboid major and intercostal muscles to provide complete early analgesic coverage. Second, because RIB primarily blocks the superficial somatic branches of the thoracic nerves, its ability to relieve visceral pain is likely limited. Early postoperative pain after thoracoscopic surgery is influenced not only by the surgical incision but also by visceral irritation, friction at the incision sites, and discomfort associated with the thoracic drainage tube. Therefore, patients receiving RIB may require supplemental systemic analgesics during the early postoperative period to compensate for its relatively limited visceral analgesic effect. In comparison, TPVB blocks the thoracic spinal nerves and sympathetic chain within the paravertebral space, providing both somatic and partial visceral analgesia, which may better control pain immediately after surgery. Further studies incorporating postoperative sensory mapping and assessment of block distribution are warranted to characterize the hypotheses underlying these differences.

Safety outcomes were comparable between the two groups, although two patients in the TPVB group developed hypotension, likely reflecting sympathetic blockade associated with paravertebral local anesthetic spread. This finding is consistent with previous reports [12]. Local anesthetic administered during TPVB may spread within the paravertebral space and through the intervertebral foramina into the epidural space, producing more extensive sympathetic blockade [18]. One patient in the RIB group developed a pneumothorax; however, it occurred contralateral to the block site in a patient with pre-existing pulmonary bullae, making a causal relationship with RIB unlikely. Because TPVB requires deeper needle advancement near the pleura and intercostal vessels, it carries a greater risk of vascular puncture, hematoma formation, pleural injury, and procedure-related pneumothorax [19]. In comparison, RIB is performed within a superficial fascial plane, avoiding direct contact with the pleura, neuraxial structures, and major vascular structures. Therefore, RIB may represent a safer regional analgesic technique for thoracoscopic surgery, particularly in patients with coagulation disorders, autonomic dysfunction, or compromised respiratory function [20].

This study has several limitations. First, a no-block control group could not be included because multimodal analgesia is the current standard of care, and withholding regional analgesia would have raised ethical concerns. Second, several other regional analgesic techniques, including the erector spinae plane block, intercostal nerve block, and serratus anterior plane block, are available for postoperative pain management after thoracoscopic surgery. Comparative studies are therefore needed to determine the relative efficacy and safety of these approaches. Third, the present investigation evaluated only a two-point injection technique; further studies are required to determine whether multi-point RIB provides additional analgesic benefits. Fourth, sensory block assessments were not performed before or after block placement, preventing accurate evaluation of block onset, dermatomal spread, and the extent of sensory coverage. Fifth, although patients, surgeons, outcome assessors, and statisticians were blinded, the anesthesiologist performing the regional block could not be blinded because of the nature of the intervention, introducing the potential for performance bias.

However, these anesthesiologists were not involved in intraoperative anesthetic management, postoperative follow-up, or outcome assessment, minimizing its potential impact. Finally, this was a single-center prospective study conducted at a tertiary center in China by anesthesiologists with substantial expertise in ultrasound-guided regional anesthesia. Therefore, the generalizability of these findings should be confirmed in future multicenter prospective studies involving more diverse patient populations and anesthesiologists with varying levels of experience.

## 6. Conclusion

In conclusion, ultrasound-guided two-point RIB provides postoperative analgesia that is non-inferior to ultrasound-guided two-point TPVB in patients undergoing three-port thoracoscopic surgery, supporting its use as an effective regional analgesic technique for this surgical procedure.

## Supplementary Material

268009373.R1-Supplementary_material_1.docx

CONSORT_2025.docx

Supplementary material 2.docx

## Data Availability

All data generated or analyzed during this study are included in this published article and its supplementary information files. The de identified individual participant data are available from the corresponding author upon reasonable request. Protocol and statistical analysis plan are provided in the Supplementary Materials 1.
