# Supplementary material for: Comparison of ultrasound-guided two-point block of the rhomboid intercostal vs. thoracic paravertebral for postoperative analgesia in patients undergoing three-port thoracoscopic surgery: a prospective, randomized, non-inferiority study
Source: Ann Med. 2026 Aug 8;58(1):2713836. doi: 10.1080/07853890.2026.2713836 (PMC13455688; doi:10.1080/07853890.2026.2713836)
Supplement: Supplementary file 1 [file IANN_A_2713836_SM0758.docx]

Supplementary Materials 2

Comparison of ultrasound-guided two-point block of the rhomboid intercostal *vs.* thoracic paravertebral for postoperative analgesia in patients undergoing three-port thoracoscopic surgery

A Prospective, Randomized, Noninferiority study

**Contents**

**S2-eFigure**

S2-eFigure 1. Thoracic paravertebral nerve block ultrasound guidance diagram. *--- 3*

S2-eFigure 2. Non-inferiority comparison of resting pain NRS scores within 48

hours after RIB and TPVB procedures (Intention-to-treat). *----------- 4*

S2-eFigure 3. Non-inferiority comparison of coughing pain NRS scores within 48

hours after RIB and TPVB procedures (Per-protocol). *-------------- 5*

S2-eFigure 4. Non-inferiority comparison of coughing pain NRS scores within 48

hours after RIB and TPVB procedures (Intention-to-treat). *--------- 6*

**S2-eTable**

S2-eTable 1. Patient characteristics, intraoperative and postoperative data

(Intention-to- treat). *----------------------------------------------------------7*

S2-eTable 2. Intraoperative and postoperative data (Per-protocol). *----------------- 8*

S2-eTable 3. Postoperative Analgesia, adverse Reactions, and complications

(Intention-to-treat). *--------------------------------------------------------- 10*

S2-eTable 4. QoR-40 score at 24 hours after surgery (Intention-to-treat). *--------- 11*

**S2-eFigure 1**. Thoracic paravertebral block ultrasound guidance diagram. TP: Transverse process; SCTL: Transverse costal ligament; TPVS: Thoracic paravertebral space; White arrow indicates local anaesthetic injection site.

**S2-eFigure 2.** Non-inferiority comparison of resting pain NRS scores within 48 hours after RIB and TPVB procedures (Intention-to-treat). RIB: Rhomboid intercostal block, TPVB: Thoracic paravertebral block, Δ: Non-inferiority margin, NRS: Numeric rating scale, CI: Confidence interval.

**S2-eFigure 3.** Non-inferiority comparison of coughing pain NRS scores within 48 hours after RIB and TPVB procedures (Per-protocol). RIB: Rhomboid intercostal block, TPVB: Thoracic paravertebral block, Δ: Non-inferiority margin, NRS: Numeric rating scale, CI: Confidence interval.

**S2-eFigure 4.** Non-inferiority comparison of coughing pain NRS scores within 48 hours after RIB and TPVB procedures (Intention-to-treat). RIB: Rhomboid intercostal block, TPVB: Thoracic paravertebral block, Δ: Non-inferiority margin, NRS: Numeric rating scale, CI: Confidence interval.

**S2-eTable 1.** Patient characteristics, intraoperative and postoperative data (Intention-to-treat)

|  | RIB(*n*=35) | TPVB(*n*=35) |
| --- | --- | --- |
| Age, y | 57.83 ± 14.03 | 57.43 ± 12.73 |
| Height, cm | 160.0(155.5-168.0) | 160.0 (156.5, 162.5) |
| Weight, kg | 62.50 (53.25, 66.75) | 58.10 (54.00, 65.50) |
| Gender，Male/Female | 13/22 | 7/28 |
| Lung surgery history, n% | 4(11.43%) | 3(8.57%) |
| ASA, n% |  |  |
| Ⅰ | 1(2.86%) | 0(0.0%) |
| Ⅱ | 31(88.57%) | 34(97.14%) |
| Ⅲ | 3(8.57%) | 1(2.86%) |
| Pathology. n% |  |  |
| Malignant | 31(88.57%) | 34(91.14%) |
| Benign | 4(11.43%) | 1(2.86%) |
| Comorbidities. n% |  |  |
| Hypertension | 13 (37.14%) | 12 (34.29%) |
| Diabetes | 4 (11.43%) | 3 (8.57%) |
| Stroke | 1(2.9%) | 1(2.9%) |
| Immunometabolic | 3 (8.57%) | 2 (5.71%) |
| COPD | 2 (5.71%) | 0 (0.00%) |
| Liver disease | 1 (2.86%) | 2 (5.71%) |
| Depression | 1 (2.86%) | 2 (5.71%) |
| Surgical side, Left /Right | 17/18 | 16/19 |
| Anesthesia, median (IQR), min | 100.0 (90.5, 117.5) | 110.0 (95.0, 130.0) |
| Surgery, median (IQR), min | 85.00 (76.00, 100.00) | 95.00 (80.00, 115.00) |
| Extubation, median (IQR), min | 24.00 (15.00, 31.50) | 25.00 (21.00, 32.50) |
| Liquid, median (IQR), ml | 1000.0(1000.0-1000.0) | 1000(1000.0-1375.0) |
| Vasoactive drugs, n% | 14 (41.18%) | 16 (47.06%) |
| Sufentanil, μg | 31.25 (26.62, 33.38) | 29.05 (27.00, 32.75) |
| Remifentanil, μg | 1088 (964.5, 1402.5) | 1221 (1020.0, 1369.5) |
| Sevoflurane, mL | 14.40 (13.03, 16.92) | 15.84 (13.68, 18.72) |
| Propofol, mg | 644.0 (582.3, 801.3) | 710.5 (610.0, 784.8) |
| ICU admission after surgery, n% | 1 (2.86%) | 2 (5.71%) |
| Hospital stay after surgery, median (IQR), days | 6.29 ± 2.78 | 6.20 ± 4.60 |
| Duration of postoperative mechanical ventilation, median (IQR), min | 26.66 ± 17.09 | 28.46 ± 14.92 |

Notes: ASA, American Society of Anesthesiologists; COPD, chronic obstructive pulmonary disease

**S2-eTable 2.** Intraoperative and postoperative data (Per-protocol)

|  | RIB(n=34) | TPVB(n=34) | *P* value |
| --- | --- | --- | --- |
| Surgical side, Left /Right | 17/17 | 15/19 | 0.627 |
| Anesthesia, median (IQR), min | 100.0(90.3-119.0) | 108.0(95.0-130.0) | 0.349 |
| Surgery, median (IQR), min | 90.4(75.0-100.0) | 99.7(80.0-115.0) | 0.155 |
| Liquid, median (IQR), ml | 1000.0(1000.0-1000.0) | 1 000(1000.0-1375.0) | 0.638 |
| Vasoactive drugs, n% | 14(41.2%) | 16(47.1%) | 0.625 |
| Anesthetic drugs, median (IQR) |  |  |  |
| Sufentanil, μg | 31.4(26.90-33.7) | 29.5(27.0-33.4) | 0.632 |
| Remifentanil, μg | 1114.0(962.0-1439.0) | 1206.0(1020.0-1372.0) | 0.371 |
| Sevoflurane, mL | 14.4(13.0-17.1) | 15.5(13.7-18.7) | 0.349 |
| Propofol, mg | 657.0(581.0-819.0) | 703.0(610.0-786.0) | 0.371 |
| Duration of postoperative mechanical ventilation, median (IQR), min | 27.44 ± 16.70 | 27.94 ± 14.82 | 0.897 |
| Hospital stay postoperative, median (IQR), days | 5.91 ± 1.71 | 6.29 ± 4.64 | 0.653 |
| ICU admission after surgery， n% | 0 (0.00%) | 2 (5.88%) | 0.473 |

Note: Vasoactive drugs include noradrenaline, norepinephrine and ephedrine.

**S2-eTable 3.** Postoperative Analgesia, adverse Reactions, and complications (Intention-to-treat)

|  | RIB (*n*=35) | TPVB (*n*=35) | *P* value |
| --- | --- | --- | --- |
| Sufentanil, median (IQR), μg | 16.00 (8.00, 19.00) | 12.00 (8.00, 20.00) | 0.723 |
| Rescue analgesia, n% | 12 (34.29%) | 4 (11.43%) | 0.046 |
| Postoperative complications, n% | 7 (20.00%) | 12 (34.29%) | 0.179 |
| PONV | 6 (17.14%) | 8 (22.86) | 0.550 |
| Hypotension | 0 (0.00%) | 2 (5.71%) | 0.473 |
| Chills | 1 (2.86%) | 4 (11.43%) | 0.353 |
| PPCs, n% | 17 (48.57%) | 14 (40.00%) | 0.470 |
| Pulmonary infection | 11 (31.43%) | 8 (22.86%) | 0.420 |
| Pleural effusion | 2 (5.71%) | 5 (14.29%) | 0.426 |
| Atelectasis | 7 (20.00%) | 5 (14.29%) | 0.526 |
| Hypoxaemia | 1 (2.86%) | 1 (2.86%) | 0.999 |
| Pneumothorax | 1 (2.86%) | 0 (0.00%) | 0.999 |
| Pneumonia | 0 (0.00%) | 2 (5.71%) | 0.473 |
| Pulmonary embolism | 0 (0.00%) | 1 (2.86%) | 0.999 |

Note: PONV, postoperative nausea and vomiting; PPCs, postoperative pulmonary complications. Pulmonary complications within 7 days post-operation included pneumothorax, pleural effusion (>300ml), atelectasis, hypoxaemia, pulmonary infection, pneumonia, and pulmonary embolism.

**S2-eTable 4**. QoR-40 score at 24 hours after surgery (Intention-to-treat)

|  | RIB (*n* = 35) | TPVB (*n* = 35) | *P* value |
| --- | --- | --- | --- |
|  |  |  |  |
| QoR-40 score at 24 hours after surgery | 179.80 ± 6.80 | 180.26 ± 6.23 | 0.770 |
| Emotions | 40.00 (40.00, 41.00) | 40.00 (40.00, 41.00) | 0.705 |
| Physical comfort | 55.00 (52.00, 55.00) | 55.00 (51.50, 55.00) | 0.533 |
| Physical independence | 21.00 (20.00, 22.00) | 21.00 (20.00, 23.00) | 0.798 |
| Pain | 32.00 (30.00, 33.00) | 33.00 (30.00, 33.00) | 0.465 |
| Psychological support | 34.00 (33.00, 34.50) | 34.00 (33.00, 35.00) | 0.487 |

Note: QoR-40, Quality of Recovery-40.
