# Supplementary material for: Comparison of ultrasound-guided two-point block of the rhomboid intercostal vs. thoracic paravertebral for postoperative analgesia in patients undergoing three-port thoracoscopic surgery: a prospective, randomized, non-inferiority study
Source: Ann Med. 2026 Aug 8;58(1):2713836. doi: 10.1080/07853890.2026.2713836 (PMC13455688; doi:10.1080/07853890.2026.2713836)
Supplement: Supplementary file 3 [file IANN_A_2713836_SM0760.docx]

Supplementary Materials 1

Trial Protocol and Statistical Analysis Plan

**Comparison of ultrasound-guided two-point block of the rhomboid intercostal vs. thoracic paravertebral for postoperative analgesia in patients undergoing three-port thoracoscopic surgery: a prospective, randomized, non-inferiority study**

**Research protocol: part 1**

**General information**

Protocol title: Comparison of ultrasound-guided two-point block of the rhombic intercostal vs. thoracic paravertebral for postoperative analgesia in patients undergoing three-port thoracoscopic surgery

Funder: Qinghe Zhou, PhD. The second affiliated hospital of Jiaxing University. No.1518, Northern Road Surrounding the City, Jiaxing City, Zhejiang Province, China

Principal Investigator: Qinghe Zhou, MD

Email Contact: jxxmxy@163.com

Co-Investigators: Mingzi An, MD, Yueru Hou, MD, Chengyun Xu, MD, Zhenping Li, MD, Yazhi Xi, MD, Xiaoyu Jia, MD, Lei Xie, MD

Corresponding author: Qinghe Zhou, PhD

Research Institutions: Affiliated hospital of Jiaxing University, Jiaxing, Zhejiang Province, China.

Tel +86-13732573379

Time frame: The project is expected to be completed between September 1, 2023 and September 1, 2025.

**Rationale & background information**

Video-assisted thoracoscopic surgery (VATS) has gained widespread acceptance in the field of thoracic surgery due to its minimally invasive nature, which significantly reduces trauma compared to traditional open thoracotomy techniques. ^1,2^ This innovative approach allows for enhanced surgical visualization through the use of multiport access, facilitating intricate procedures with greater precision. However, despite these advantages, patients often experience considerable postoperative pain, particularly during the early stages of recovery when they attempt to mobilize. This pain can hinder the overall recovery process and prolong hospital stays, necessitating effective pain management strategies. ^3,4^ Regional nerve blocks have emerged as a valuable tool in perioperative pain management, offering rapid and sustained analgesic effects that can significantly alleviate discomfort following thoracic procedures. By targeting specific nerve pathways, these blocks can minimize the reliance on opioids, thereby reducing the risk of opioid-related adverse effects, such as nausea, constipation, and potential dependency. The implementation of regional anesthesia not only enhances patient comfort but also promotes a more efficient postoperative recovery, enabling patients to engage in early mobilization and rehabilitation. Consequently, the integration of regional nerve blocks in conjunction with VATS represents a promising advancement in optimizing patient outcomes in thoracic surgery.

Thoracic epidural anaesthesia (TEA) has long been regarded as the gold standard for managing post-thoracic surgical pain, offering substantial analgesic benefits. However, the associated side effects of TEA, including potential complications such as hypotension and urinary retention, have raised concerns regarding its alignment with the principles of enhanced recovery after surgery (ERAS). In light of these challenges, thoracic paravertebral block (TPVB) has emerged as a promising alternative, demonstrating effective analgesia for patients undergoing thoracoscopic procedures. TPVB may indeed supplant TEA as the primary modality for post-thoracoscopic surgical pain management, given its potential to minimize systemic side effects and promote faster recovery. ^5^ Nevertheless, the application of TPVB in the context of three-thoracoscopic postoperative analgesia remains a subject of ongoing debate within the medical community. The technique necessitates precise needle placement in close proximity to the pleural space, which inherently carries risks. Complications such as hypotension, chest wall hematoma, pneumothorax, and the inadvertent onset of epidural anesthesia can occur, necessitating careful consideration and skilled execution of the procedure. As the search for optimal analgesic strategies continues, further research is essential to establish the safety and efficacy of TPVB in this specific surgical context. ^6^

An innovative technique within the realm of chest wall plane blocks, the rhomboid intercostal block (RIB) provides coverage of the surgical incision area and puncture point that is distant from the thoracic cavity and major blood vessels. ^7–9^ Our previous studies have indicated that the analgesic effect of single-shot RIB after thoracoscopic surgery is non-inferior to that of single-shot TPVB.^10^ However, three-port thoracoscopic surgery necessitates incisions spanning multiple segments, with the efficacy of a singular regional block for pain relief remaining ambiguous.

We hypothesized that the ultrasound-guided two-point injection RIB might provide analgesia and postoperative recovery quality as effective as that of ultrasound-guided two-point injection TPVB for three-port thoracoscopic surgery.

**Methods**

**Study participants**

According to the study protocol, patients were enrolled (S1-eFigure 1 and S1-eTable 1). The study inclusion criteria were (1) single-sided three-port thoracoscopic surgery; (2) age 18-80 years; (3) ASA grade I-III. The exclusion criteria were (1) cardiac, pulmonary, hepatic, renal and coagulation function abnormalities (LVEF%≤40%, PaO₂/FiO₂ ratio ≥ 300mmHg, Child-Pugh C, creatinine ≥ 133 μmol/L, and INR ≥ 1.2); (2) planned blockade region infection, severe nerve injury on the blockade side limb; (3) patients with a history of chronic pain medication, psychiatric drug use, or contraindications to non-steroidal anti-inflammatory drugs; (4) patients allergic to local anaesthetics such as lidocaine, ropivacaine, or general anaesthetic agents; (5) patients or relatives refusing surgery or anaesthesia; (6) patients unable to complete the questionnaire.

The elimination criteria include (1) patients experiencing serious complications during surgery; (2) patients with significant blood loss during surgery (greater than 800 ml); (3) patients refusing follow-up; (4) patients whose surgery was converted to open thoracotomy or single-port thoracoscopic surgery.

**General anesthesia and postoperative management**

The anesthesia protocol involved a combination of intravenous and inhalational agents. Anesthetic induction included rocuronium at a dose of 0.6 mg/kg, sufentanil at 0.4–0.8 μg/kg, propofol at 2 mg/kg, and atropine at 0.5 mg. Maintenance of anesthesia consisted of propofol at a rate of 50–150 μg/(kg·min), remifentanil at 0.1–1 μg/(kg·min), and inhaled sevoflurane at 1–1.5 MAC. Rocuronium boluses of 0.15 mg/kg were intermittently administered during surgery to ensure adequate muscle relaxation based on drug kinetics and clinical requirements. The choice of a 37^#^ or 35^#^ double-lumen endotracheal tube was made based on the patient's sex. Both patient groups were subjected to volume-controlled mode mechanical ventilation during the procedure, with ventilation parameters set at Vt 6-8 ml/kg, RR 10-16 breaths/min, and PetCO2 maintained between 35–45 mmHg. Anesthetic depth was adjusted based on the Entropy Sensor (GE Healthcare Finland Oy). Fluid replacement adhered to the 4-2-1 rule to maintain circulatory stability within the normal range, while vasoactive drugs were administered in response to specific intraoperative hemodynamic conditions.

During skin closure, the patient undergoing surgery received ondansetron 4 mg to prevent post-operative nausea and vomiting. Subsequently, the patient was connected to a patient-controlled intravenous analgesia pump containing sufentanil 100 μg in 0.9% sodium chloride solution (diluted to 100 ml). The pump was set with a bolus dose of 2 ml per press, a lockout time of 15 minutes, an initial dose of 2 ml, and no background infusion. If, within 48 hours postoperative, the patient pressed the analgesia pump and reported an acute numerical rating scale (NRS) score of ≥4, parecoxib 40 mg was administered as rescue analgesia.

**Regional blocks**

TPVB group: Initially, the ultrasound probe is positioned vertically at the T5 level along the midline of the trunk, employing a linear ultrasound probe (LOGIQe, GE ultrasound equipment; Deutschland GmbH & Co. KG, Solingen, Germany) operating at a frequency of 10 MHz. The first step is to identify the spinous process, which serves as a critical landmark for subsequent maneuvers. Following this, the ultrasound probe is carefully maneuvered towards the surgical side, allowing visualization of essential anatomical structures including the transverse process, pleura, and intercostal membrane. Once the appropriate structures are identified, the needle is inserted in alignment with the ultrasound probe plane. Real-time ultrasound imaging facilitates the visualization of the needle trajectory, ensuring precision as the needle tip enters the paravertebral space, ultimately reaching the base of the transverse process. At this juncture, a careful injection of 10 ml of 0.5% ropivacaine hydrochloride is administered into the paravertebral space. The effectiveness of the injection is confirmed by observing pleural indentation and the subsequent spread of the local anesthetic within the paravertebral space. This technique is then replicated at the T7 level, adhering to the same methodical approach to ensure consistent and effective nerve blockade.

RIB group: Patient positioned in left or right lateral decubitus position, with the ipsilateral arm passed through and adducted across the chest, scapula deliberately moved laterally to optimize anatomical visualization. High-resolution linear ultrasound probe positioned at 10 MHz frequency on an oblique sagittal plane immediately medial to the scapular medial border. Careful identification of key ultrasound anatomical markers including superficial trapezius muscle, deeper rhomboid major muscle, underlying intercostal muscles, parietal pleura, and lung tissue interface. Using a precise in-plane needle approach, needle inserted horizontally between thoracic vertebrae T4 and T5, with meticulous needle manipulation involving controlled withdrawing and advancing techniques. After initial nerve blockade visualization, needle precisely positioned within fascial interval separating rhomboid major muscle and intercostal muscles. Initial test injection of 0.5% ropivacaine at 0.5-1 ml volume, carefully observing local anesthetic spread and confirming correct fascial plane location. Following confirmation, definitive local anesthetic injection of 10 ml 0.5% ropivacaine into identified fascial plane interval. Identical blockade procedure systematically repeated at horizontal level between thoracic vertebrae T6 and T7 using identical technical approach and methodology.

**Randomization and blinding**

A random sequence was generated by a researcher unrelated to this study using block randomisation with a 1:1 allocation ratio, with block sizes of 4 before patients enrolled in the study. Random numbers were placed in numbered opaque envelopes. On the day of surgery, another researcher opened the envelope and allocated each patient to the corresponding study group according to the numbers inside. All patients, surgeons, research statisticians and outcome assessors were blinded to group allocation.

Nerve blockade was administered by an experienced anesthetist who was not involved in postoperative follow-up and analysis, utilizing ultrasound guidance in the pre-anesthesia preparation area.

**Data Collection and Outcome Assessment**

Data pertaining to patient characteristics, surgical procedures, and anesthesia were obtained from the electronic anesthesia records or the patient’s clinical notes.

The primary outcome was the NRS pain score at rest 24h postoperative. Secondary outcomes included NRS scores for rest and coughing at 0.5, 2, 4, 12, 18, and 48 h (including coughing NRS score at 24 hours), QoR-40 score at 24h postoperation, total sufentanil usage at 48h postoperation, and the necessity of rescue analgesia postoperatively.

NRS: The Numerical Rating Scale uses a range from 0 to 10 to represent different levels of pain, with 0 indicating no pain and 10 indicating extreme pain. The pain intensity grading criteria are as follows: 0: no pain; 1-3: mild pain; 4-6: moderate pain; 7-10: severe pain. The QoR-40 scoring system assesses patient recovery from five aspects: emotional state (9 questions), physical comfort (12 questions), self-care ability (5 questions), psychological support (7 questions), and pain (7 questions). Each question is scored on a scale of 5 points, with a maximum score of 200 points. A higher score indicates a better recovery status (S1-eTable 2).

**Safety considerations**

A adverse event is any adverse drug or biologic or device experience occurring at any dose that results in any of the following outcomes: (1) Safety outcomes at 24h postoperative included postoperative nausea and vomiting (PONV), hypotension, chest wall haematoma, respiratory depression, shivering, urinary retention, accidental epidural anaesthesia; (2) Pulmonary complications within 7 days post-operation included pneumothorax, pleural effusion (>300ml), atelectasis, hypoxaemia, pulmonary infection, pneumonia, and Pulmonary embolism (S1-eTable 3).

**Problems anticipated**

Prior to conducting the anesthesia and surgery procedures, it is imperative for the researchers to acquire the consent of the thoracic surgeon and apprise them of the pertinent steps involved. The procurement of drugs and inhalation equipment required for the study necessitates the submission of a request for funding. The present investigation received financial backing from Zhejiang Medical and Health Science and Technology plan project (2021KY399), and the Zhejiang Province Clinical Key Specialty Construction Project - Anesthesiology Department (2023-ZJZK-001).

**Dissemination of results and publication policy**

Experimental design and manuscript writing: Mingzi An; Research implementation, data collection: Yueru Hou, Chengyun Xu; Nerve Block: Zhenping Li; Folllow-up: Lei Xie; Statistical analysis: Xiaoyu Jia; Funding support, research Supervision and manuscript revision: Qinghe Zhou.

**Ethics**

The study received approval from the Ethics Committee of the Affiliated Hospital of Jiaxing University (2023-KY-340) and successfully registered with the Chinese Clinical Trial Registry (ChiCTR2300073661).

**Research protocol: part 2**

**Budget**

The study budget has been allocated as 10,000 RMB for the procurement of drugs and inhalation devices for the clinical trial.

**Other support for the project**

Not applicable.

**Collaboration with other scientists or research institutions**

Not applicable.

**Links to other projects**

Not applicable.

**Other research activities of the investigators**

Not applicable.

**Financing and insurance**

Not applicable.

**Data management and statistical analysis**

Sample Size Estimation

A pilot study (n=20, unpublished data) showed that the mean 24h resting NRS were 2.40 ± 0.70 for RIB block and 2.30 ± 1.25 for TPVB (control group). Statisticians and clinical experts generally recommend setting the non-inferiority margin (Δ) as 20% to 50% of the standard deviation of the control group. The minimal clinically important difference (MCID) for postoperative acute pain assessed by VAS or NRS is 1 point. ^11^ Previous non-inferiority trials investigating analgesia after thoracoscopic surgery adopted a non-inferiority margin of 1. ^12^ Therefore, we defined our acceptable non-inferiority margin as Δ=1. For a two-independent-sample non-inferiority test of means, 56 evaluable patients were required to achieve 90% statistical power with a one-sided α of 0.025. A total of 70 patients were planned for enrollment to account for an anticipated 20% dropout rate.

**Statistical analysis**

All patients randomized were analyzed according to the intention-to-treat principle (ITT), and the per-protocol patients were employed for the per-protocol analysis (PP). Non-inferiority is determined by comparing the lower limit of the 95% confidence interval (95% CI) of the mean difference between groups at rest and coughing within 48 hours on the NRS scale, with a Δ of 1. The meeting of non-inferiority criteria in this study is defined as a lower limit of the 95% CI of the mean 24-hour resting NRS difference between groups that does not exceed the Δ of 1. Descriptive statistics were utilized for analyzing baseline characteristics. Continuous variables with a normal distribution were presented as mean ± standard deviation, while non-normally distributed data were expressed as median (interquartile range). Categorical variables were reported as number (%). Intergroup variances were evaluated using independent samples t-test or Mann-Whitney U test, and count data were assessed using chi-squared test or Fisher's exact test. Statistical analyses were conducted using SPSS 25.0, and graphs were generated using Graphpad Prism 9. A significance level of *P*<0.05 was adopted.

**Quality assurance**

The research Team will consist of one mentor and three students, and the mentor not directly involved with the study. The research team will periodically review the study data and, according to the protocol, determine whether it is safe to continue the study. The research team has access to all data available in the study throughout the study period. All serious adverse events (SAEs), including deaths, will be reported immediately to the study team. The purpose of the research team is to ensure safety.

For each patient enrolled, a CRF must be completed and signed by the principal investigator or authorized delegate from the study staff. This also applies to records for those patients who fail to complete the study. If a patient withdraws from the study, the reason must be noted on the CRF. If a patient is withdrawn from the study because of a treatment-limiting adverse event, thorough efforts should be made to clearly document the outcome. The investigator should ensure the accuracy, completeness, legibility and timeliness of the data in the CRFs and in all required reports.

**References**

1.	Bendixen M, Jørgensen OD, Kronborg C et al, Postoperative pain and quality of life after lobectomy via video-assisted thoracoscopic surgery or anterolateral thoracotomy for early stage lung cancer: a randomised controlled trial. *The Lancet Oncology* 2016; 17:836–44

2.	Wang H, Li S, Liang N, et al, Postoperative pain experiences in Chinese adult patients after thoracotomy and video-assisted thoracic surgery. *J Clin Nurs* 2017; 26:2744–54

3.	Umari M, Carpanese V, Moro V, et al. Postoperative analgesia after pulmonary resection with a focus on video-assisted thoracoscopic surgery. *Eur J Cardiothorac* Surg 2018; 53:932–8

4.	Feray S, Lubach J, Joshi GP, et al. PROSPECT guidelines for video‐assisted thoracoscopic surgery: a systematic review and procedure‐specific postoperative pain management recommendations. *Anaesthesia* 2022; 77:311–25

5.	Yeung JH, Gates S, Naidu BV, et al, Paravertebral block versus thoracic epidural for patients undergoing thoracotomy. *Cochrane Database Syst Rev* 2016; 2016:CD009121

6.	Ardon AE, Lee J, Franco CD, et al, Paravertebral block: anatomy and relevant safety issues. *Korean J Anesthesiol* 2020; 73:394–400

7.	Deng W, Liu F, Jiang C, et al, Continuous Rhomboid Intercostal Block for Thoracoscopic Postoperative Analgesia. *The Annals of Thoracic Surgery* 2022; 114:319–26

8.	West D, Evaluation of rhomboid intercostal and subserratus plane block under direct vision for postoperative analgesia in thoracic surgeries: a prospective, randomized controlled trial, thoracic non-oncologic. *Eur J Cardiothorac Surg* 2022; 62:ezac532

9.	Kozanhan B, Semerkant T, Esme H, et al, Evaluation of rhomboid intercostal and subserratus plane block under direct vision for postoperative analgesia in thoracic surgeries: a prospective, randomized controlled trial. *Eur J Cardiothorac Surg* 2022; 62:ezac498

10.	Wang X, Jia X, Li Z, et al, Rhomboid intercostal block or thoracic paravertebral block for postoperative recovery quality after video-assisted thoracic surgery. *Eur J Anaesthesiol* 2023; 40:652–9

11.	Myles PS, Myles DB, Galagher W, et al. Measuring acute postoperative pain using the visual analog scale: the minimal clinically important difference and patient acceptable symptom state. *Br J Anaesth*. 2017;118(3):424-429.

12. Taketa Y, Irisawa Y, Fujitani T. Comparison of ultrasound-guided erector spinae plane block and thoracic paravertebral block for postoperative analgesia after video-assisted thoracic surgery: a randomized controlled non-inferiority clinical trial. *Reg Anesth Pain Med* 2019; rapm-2019-100827.

**Figure Legend**

**Figure 1.** Study Protocol Flowchart

**S1-eFigure 1.** Study Protocol Flowchart

**S1-Table 1.** Duration of the project

| 04/2023-09/2023 | Formulate the research protocol, formulate screening criteria, complete the preliminary experiment, and calculate sample size. |
| --- | --- |
| 09/2023-09/2024 | Collect data, analyze data and write papers. |
| 10/2025-12/2026 | Collation of protocol programs and publication of papers. |

**S1-Table 2.** Definition of postoperative pulmonary complications.

| Respiratory infection | Antibiotics for suspected infection with one or more of the following: new or changed sputum, new or changed lung opacities, fever, white blood cell count >12 × 10^9^ litre^-1^. |
| --- | --- |
| Pulmonary embolism | A clinical syndrome characterized by the obstruction of the pulmonary artery and its branches by endogenous or exogenous emboli, leading to pulmonary circulation and right heart dysfunction. Common types of emboli include thrombi, fat, amniotic fluid, air, and tumor cells. |
| Pleural effusion | CXR with blunting of costophrenic angle, loss of sharp silhouette of the ipsilateral hemidiaphragm in upright position, displacement of adjacent anatomical structures, or (in supine position) hazy opacity in one hemithorax with preserved vascular shadows. |
| Atelectasis | Lung opacification with mediastinal shift, hilum or hemidiaphragm shift towards the affected area, with compensatory hyperinflation in adjacent non-atelectatic lung. |
| Pneumonia | CXR with at least one of the following: infiltrate, consolidation, cavitation; plus at least one of the following: fever >38℃ with no other cause, white cell count <4 or >12 × 10^9^ litre^-1^, >70 yr of age with altered mental status with no other cause; plus at least two of the following: new purulent/changed sputum, increased secretions/suctioning, new/worse cough/dyspnoea/tachypnoea, rales/ bronchial breath sounds, worsening gas exchange. |
| Pneumothorax | Air in the pleural space with no vascular bed surrounding the visceral pleura |
| Hypoxemia | Ventilated, bilateral infiltrates on CXR, PaO_2_ :FIO_2_ ≤300, minimal evidence of left atrial fluid overload within 7 days of surgery. |

**S1-Table 3.** QoR-40 Assessment Scale

**Part 1**

How have you felt in the past 24 hours? (This section uses a 1-5 scoring system. A higher score indicates that the feeling was present more often in the past 24 hours. 1 = Never, 5 = All of the time)

**I. Physical Comfort
(1 Never; 2 A little of the time; 3 Some of the time; 4 Most of the time; 5 All of the time)**

| 1.Able to breathe easily | 1 | 2 | 3 | 4 | 5 |
| --- | --- | --- | --- | --- | --- |
| 2. Had a good sleep | 1 | 2 | 3 | 4 | 5 |
| 3. Had a good appetite | 1 | 2 | 3 | 4 | 5 |
| 4. Felt rested and energetic | 1 | 2 | 3 | 4 | 5 |

**Total Score:**

**II. Emotional State
(1 Never; 2 A little of the time; 3 Some of the time; 4 Most of the time; 5 All of the time)**

| 1. Felt generally well | 1 | 2 | 3 | 4 | 5 |
| --- | --- | --- | --- | --- | --- |
| 2. Able to control emotions | 1 | 2 | 3 | 4 | 5 |
| 3. Felt happy and relaxed | 1 | 2 | 3 | 4 | 5 |

**Total Score:**

**III. Social Support
(1 Never; 2 A little of the time; 3 Some of the time; 4 Most of the time; 5 All of the time)**

| 1. Able to communicate well with medical staff | 1 | 2 | 3 | 4 | 5 |
| --- | --- | --- | --- | --- | --- |
| 2. Able to communicate well with family/friends | 1 | 2 | 3 | 4 | 5 |
| 3. Felt supported by doctors | 1 | 2 | 3 | 4 | 5 |
| 4. Felt supported by nurses | 1 | 2 | 3 | 4 | 5 |
| 5. Felt supported by family/friends | 1 | 2 | 3 | 4 | 5 |
| 6. Able to understand instructions/advice from others | 1 | 2 | 3 | 4 | 5 |

**Total Score:**

**IV. Physical Independence
(1 Never; 2 A little of the time; 3 Some of the time; 4 Most of the time; 5 All of the time)**

| 1. Able to engage in usual work/household activities | 1 | 2 | 3 | 4 | 5 |
| --- | --- | --- | --- | --- | --- |
| 2. Able to write | 1 | 2 | 3 | 4 | 5 |
| 3. Able to speak normally | 1 | 2 | 3 | 4 | 5 |
| 4. Able to wash/shave independently | 1 | 2 | 3 | 4 | 5 |
| 5. Able to groom oneself | 1 | 2 | 3 | 4 | 5 |

**Total Score:**

**Part 2**

**Have you experienced any of the following in the past 24 hours? (This section is scored from 5 to 1, where 5 = Never, 1 = All of the time)**

**I. Discomfort
(5 Never; 4 A little of the time; 3 Some of the time; 2 Most of the time; 1 All of the time)**

| 1. Nausea | 1 | 2 | 3 | 4 | 5 |
| --- | --- | --- | --- | --- | --- |
| 2. Vomiting | 1 | 2 | 3 | 4 | 5 |
| 3. Retching | 1 | 2 | 3 | 4 | 5 |
| 4. Felt restless | 1 | 2 | 3 | 4 | 5 |
| 5. Shaking/trembling | 1 | 2 | 3 | 4 | 5 |
| 6. Shivering | 1 | 2 | 3 | 4 | 5 |
| 7. Felt very cold | 1 | 2 | 3 | 4 | 5 |
| 8. Felt dizzy or lightheaded | 1 | 2 | 3 | 4 | 5 |

**Total Score:**

**II. Emotional Distress
(5 Never; 4 A little of the time; 3 Some of the time; 2 Most of the time; 1 All of the time)**

| 1. Had nightmares | 1 | 2 | 3 | 4 | 5 |
| --- | --- | --- | --- | --- | --- |
| 2. Felt anxious | 1 | 2 | 3 | 4 | 5 |
| 3. Felt irritable | 1 | 2 | 3 | 4 | 5 |
| 4. Felt depressed | 1 | 2 | 3 | 4 | 5 |
| 5. Felt lonely | 1 | 2 | 3 | 4 | 5 |
| 6. Had difficulty falling asleep | 1 | 2 | 3 | 4 | 5 |

**Total Score:**

**III. Social & Cognitive
(5 Never; 4 A little of the time; 3 Some of the time; 2 Most of the time; 1 All of the time)**

| 1. Felt confused or disoriented | 1 | 2 | 3 | 4 | 5 |
| --- | --- | --- | --- | --- | --- |

**Total Score:**

**IV. Pain
(5 Never; 4 A little of the time; 3 Some of the time; 2 Most of the time; 1 All of the time)**

| 1. Moderate pain | 1 | 2 | 3 | 4 | 5 |
| --- | --- | --- | --- | --- | --- |
| 2. Severe pain | 1 | 2 | 3 | 4 | 5 |
| 3. Headache | 1 | 2 | 3 | 4 | 5 |
| 4. Muscle aches | 1 | 2 | 3 | 4 | 5 |
| 5. Back pain | 1 | 2 | 3 | 4 | 5 |
| 6. Sore throat | 1 | 2 | 3 | 4 | 5 |
| 7. Mouth sores | 1 | 2 | 3 | 4 | 5 |

**Total Score:**
